# Proinflammatory profile of neonatal monocytes induced by microbial ligands is downmodulated by histamine

**DOI:** 10.1038/s41598-019-50227-8

**Published:** 2019-09-23

**Authors:** Anna Cláudia Calvielli Castelo Branco, Nátalli Zanete Pereira, Fábio Seiti Yamada Yoshikawa, Luanda Mara da Silva Oliveira, Franciane Mouradian Emidio Teixeira, Luana de Mendonça Oliveira, Anna Julia Pietrobon, Marina Passos Torrealba, Josenilson Feitosa de Lima, Alberto José da Silva Duarte, Maria Notomi Sato

**Affiliations:** 1 Laboratory of Medical Investigation, LIM-56, Department of Dermatology, Tropical Medicine Institute of São Paulo, Medical School, University of São Paulo, São Paulo, Brazil; 2 Department of Immunology, Institute of Biomedical Sciences, University of São Paulo, São Paulo, Brazil

**Keywords:** Inflammation, Toll-like receptors

## Abstract

Although the neonatal period is characterized by relative immunological immaturity, an inflammatory response due to Toll-like receptor (TLR) activation is observed. Histamine may be one of the factors playing a role in restraining inflammation during the early stages of life. Therefore, we evaluated the responsiveness of human cord blood cells to TLR4 agonists and the immunomodulatory function of histamine in the inflammatory response. Compared with adults, mononuclear cells (MNCs) from newborns (NBs) exhibit impaired production of IFN-γ-inducible chemokines, such as CXCL10 and CXCL9, upon lipopolysaccharide (LPS) stimulation. Notably, LPS induced a 5-fold increase in CCL2 secretion in NBs. Evaluation of the effect of histamine on LPS-induced CCL2 secretion showed an inhibitory effect in the majority of adults, whereas this effect was detectable in all NBs. Histamine receptor (HR) blockage revealed partial involvement of H1R, H2R and H4R in LPS-induced CCL2 inhibition in MNCs from both NBs and adults. As monocytes are the main type of mononuclear cell that produces CCL2, we evaluated genes related to TLR signaling upon LPS stimulation. Monocytes from NBs showed up-regulation of genes associated with JAK/STAT/NF-κB and IFN signaling. Some differentially expressed genes encoding proinflammatory factors were preferentially detected in LPS-activated monocytes from NBs, and markedly down-regulated by histamine. The immunomodulatory role of histamine on CCL2 and CXCL8 was detected at the transcript and protein levels. Our findings show that NBs have enhanced CCL2 responsiveness to LPS, and that histamine acts in immune homeostasis during the neonatal period to counterbalance the robustness of TLR stimulation.

## Introduction

During the neonatal stage, the immune system is described to be relatively immature, because of the decreased number and function of lymphocytes and antigen-presenting cells (APCs), and the lack of cells that confer immunological memory. Despite these characteristics, neonatal immune cells exhibit a flexibility that allows them to establish a potent immune response *in vitro* depending on the type of stimulation^1,2^

Infection, including bacterial sepsis, in newborns and infants is a major health care issue^3^. Expressed by several types of cells, sentinel receptors for pathogen detection, which are referred to as pattern recognition receptors (PRRs), can recognize conserved motifs of pathogens (PAMPs, pathogen-associated molecular patterns) such as bacteria, fungi and viruses. These molecular structures include Toll-like receptors (TLRs), which initiate signaling pathways upon recognition of PAMPs, triggering inflammatory responses and maturation of the adaptive immune response^4^. In neonatal cells, TLR-mediated response can preferably lead to Th2 and Th17-type immunity instead of Th1-responses, which fight intracellular pathogens more efficiently^5^. Moreover, immune regulatory factors play important roles *in utero* in fetal-maternal tolerance and in suppressing proinflammatory responses in neonates. However, the influence of mediators that may exhibit modulatory as well as inflammatory effects, such as histamine, in early life has not yet been determined.

Histamine is a hypersensitivity mediator involved in acute inflammatory responses that has been related to chronic inflammation in several immunological events, such as hematopoiesis, cell differentiation, proliferation and regeneration^6^. Additionally, it has also been described that histamine can regulate monocytes, dendritic cells (DCs), T cells and B cells response, been involved in antibody isotype responses. This immune regulatory ability of histamine is probably associated to the differential expression and pathways triggered by its four receptors^7^. Bacteria can also secrete histamine, and elevated levels of these microorganisms in the microbiota have been associated with asthma^8^.

Because of Histamine effects on cell growth and differentiation, and vasoactive properties, this inflammatory mediator can act in embryo-uterus interactions during pregnancy. However, high levels of histamine in maternal blood have been associated with complications, such as preeclampsia, miscarriage and preterm gestation^9,10^. Curiously, the placenta highly expresses the enzyme diamine oxidase, which acts as a metabolic barrier preventing the excessive entry of bioactive histamine in the maternal or fetal blood^11^.

Histamine can drive Th2 responses by acting on DCs, contributing to the initiation and maintenance of this profile in allergic disorders^12^. In addition, histamine can promote IL-4 and inhibit IFN-γ production by modifying the actin cytoskeleton organization induced by TLR4, but not TLR2, in primed T cells^13^. It also suppresses the production of IL12p70 and CXCL10 in myeloid DCs (mDCs) after TLR2 or TLR4 stimulation^13^. Histamine may also exert a modulatory effect on activated macrophages by down-regulating histamine receptor mRNA expression, similar to the action of zymosan A in mature monocyte-derived DCs. On the other hand, alternatively activated macrophages express higher level of H2R and H4R histamine receptors^14^.

Activation of the H2R histamine receptor can negatively regulate effector T cells activation by promoting the increase of intracellular cyclic adenosine monophosphate (cAMP)^15,16^. Furthermore, as H4R histamine receptor is expressed in several immune cells and has chemoattractant properties, it strengthens histamines immunomodulatory effects in inflammatory disorders, such as allergy, asthma, chronic pruritus and autoimmune diseases^17^.

Although histamine plays roles in innate and adaptive immune responses, the impact of the immunomodulatory effect mediated by histamine on TLR-activated cells in early life has not yet been investigated. Here, we assess whether histamine can modulate the TLR4 responsiveness of neonatal monocytes by evaluating chemokine secretion and expression profiles of genes related to the TLR pathway.

## Materials and Methods

### Study population

For this study, we enrolled healthy adult individuals (n = 23; 13 females, 10 males; median age of 27.2 ± 1.5 years) and obtained cord blood from full-term mothers via cesarian section (n = 28, median age of 30.2 ± 3.2 years), recruited at São Paulo University Maternity Hospital. Peripheral blood cord was collected from the placenta at the time of delivery. All cases were negative for toxoplasmosis, HIV-1, rubella, syphilis, and hepatitis B, C. None of the individuals included in the study had asthma or rhinitis or used antihistamines. Characteristics of the newborns (NBs) at birth are shown in Table 1. This study was approved by the Ethics Committee of the University of Sao Paulo School of Medicine (07806512.0.0000.0068), and informed consent was obtained from all subjects (mothers provided consent on behalf of their newborns). This work was conducted in accordance with the Declaration of Helsinki.

**Table 1:** Characteristics of the newborns at birth.

Mother age (y)	30.2 ± 3.2
Gestational age (weeks)	39.7 ± 0.6
Newborn Apgar score	08/09/10
Gender (NBs)	13 M/15 F
NB weight (g)	3530.0 ± 128.4

n = 28; mean ± SD.

### Cell preparations

Mononuclear cells (MNCs) were isolated from cord blood and from venous blood of healthy adult individuals via Ficoll-Hypaque gradient centrifugation (GE Healthcare Bio-Sciences AB, Uppsala, Sweden). The cells were cultured in the presence of antagonists of H1R (pyrilamine, 100 μM), H2R (cimetidine, 1000 μM) and H4R (JNJ7777120, 1 μM) for 1 h and then with histamine (10 μM) (Sigma-Aldrich, St. Louis, MO, USA) and either a TLR-4 agonist (ultrapure lipopolysaccharide (LPS) from *Salmonella minnesota*, 1 μg/mL; endotoxin level ≤1 × 10^5^ EU/mg) or a dual TLR7/TLR8 agonist (CL097, 2.5 μg/mL; Invivogen, San Diego, CA, USA) for 24 h. In some experiments, human IFN-γ recombinant (25 ng/mL, Peprotech, Rock Hill, NJ, USA) was added to LPS-stimulated cultures.

### Determination of chemokines and cytokines

Chemokines were quantified using a chemokine kit, by flow cytometry (detection limits: CXCL8, 0.2 pg/mL; CXCL9, 2.8 pg/mL; CCL2, 2.7 pg/mL; CCL5, 1 pg/mL; CXCL10, 2.8 pg/mL). IFN-γ levels (3.7 pg/mL) were determined via flow cytometry using a cytometric bead array kit (BD Pharmingen, CA, USA) according to the manufacturer’s instructions. Serum cytokine levels, including IL-6 (limit of detection 68.4 fg/mL), TNF (67.3 fg/mL), IL-1β (48.4 fg/mL), IL-10 (274 fg/mL) and IL-12p70 (274 fg/mL), were detected using the enhanced sensitivity flex method. Samples were processed with a flow cytometer (LSRFortessa – BD Biosciences), and the results were used to generate graphics and tables using BD CBA Analysis Software.

### qPCR

Evaluation of histamine receptors (H1R, H2R, H3R and H4R) and histidine decarboxylase (HDC) in MNCs and for IL1B, TNF, IL6, TOLLIP, SIGRR, IFNB and TLR4 in purified monocytes from MNCs was performed via real-time polymerase chain reaction (qPCR). Total RNA was extracted using a RNeasy Plus Mini Kit (Qiagen, Valencia, CA, USA), and reverse transcription was performed with a Sensiscript Reverse Transcriptase Kit (Bio-Rad, Berkeley, CA, USA). The primers used in qPCR assay are listed in Table S1. GAPDH mRNA levels in all samples were used as internal normalizing gene. PCR was performed using an Applied Biosystems 7500 system with specific primers and SYBR Green (Applied Biosystems, Carlsbad, CA, USA) fluorescence detection reagents. Normalized and relative expression was calculated as previously described^18^. Primers were only accepted if their efficiency was 100 ± 10%. The specificity of the reaction was examined using dissociation curves.

### TLR signaling pathway PCR array

Purified monocytes were obtained from MNCs via negative selection with magnetic beads (Miltenyi Biotec, Bergish, Gladbach, Germany). Cells with a purity greater than 90%, as measured by flow cytometry, were utilized. Monocytes obtained from 3 samples from NBs and 3 samples from healthy individuals were incubated with LPS (1 µg/mL) in the presence of histamine (10 µM) or medium for 4 h at 37 °C. The cellular RNA was then extracted using the RNeasy Micro Kit (Qiagen). First-strand cDNA was synthesized based on the instructions of RT2 First-Strand and PreAMP Kit (Qiagen, Valencia, CA, USA), and the cDNA product was used for RT2 Profiler PCR Array using SYBR Green-based qPCR according to the manufacturer’s protocol (PAHS-018Z array, Qiagen). The array consisted of a panel of 84 genes related to the TLR signaling pathway, as shown in Table S2. ΔΔCt values were calculated as the difference between the Ct of TLR pathway genes and the geometric average of the Ct of housekeeping genes, ACTB (actin-beta), B2M (beta-2-microglobulin), GAPDH (glyceraldehyde 3-phosphate dehydrogenase) and HPRT1 (hypoxanthine phosphoribosyltransferase 1), in relation to the adult samples. Heatmaps were generated in R software for Mac OS X GUI (version 3.6.0) with the package “gplots” using the relative expression values normalized as follows: basal (normalized against mean adult Ct values), LPS (normalized against individual basal Ct values) and LPS histamine (normalized against individual LPS Ct values). Venn diagrams were generated in R software for Mac OS X GUI (version 3.6.0) with the package “limma” using the fold change values calculated as the newborn/adult ratio in each experimental condition. Volcano plots were generated in R software for Mac OS X GUI (version 3.6.0) with the package “ggplot2” using the fold change values calculated as the newborn/adult ratio in each experimental condition and the q-values calculated from the relative expression datasets using the False Discovery Rate (FDR) approach (Two-stage step-up method of Benjamini, Krieger and Yekutieli). The qPCR array database is available on NCBI’s GEO platform: https://www.ncbi.nlm.nih.gov/geo/query/acc.cgi?acc = GSE136478.

### Flow cytometry

To analyse intracellular staining in purified monocytes, staining was performed using the following antibodies: CD3-BV605 (SK7), CD19-Horizon V500 (HIB19, CD16-FITC (3G8), CD14-Horizon V450 (MØP9), IL-10-APC (JES3-19F1), CXCL8 –PE (G265-8), CCL2-PE (2H5, e-Bioscience, Thermofisher, Hwalthan, Massuchsetts, EUA). Antibodies were from BD Biosciences (San Jose, CA, USA). Monocytes stimulated with LPS or LPS plus histamine were incubated with Brefeldin A (10 µg/mL) for 18 hrs. Monocytes were washed and incubated with a LIVE/DEAD Fixable Red Dead Cell Stain kit (Invitrogen, Carlsbad, CA, USA) for 30 min at room temperature. Afterwards cells were blocked with human immunoglobulin for 10 min at 4 °C. This was followed by extracellular staining with antibodies for 30 min at 4 °C, fixed with a formaldehyde-0.4% (Sigma) and permeabilized with saponin-0.05% (Merck, Darmstadt, Germain). Staining was performed for intracellular molecules and surface markers for 30 min at 4 °C. After washing with isotonic solution (Hemoton SPEC; São Paulo, Brazil), 150,000 events were acquired using a flow cytometer (LRSFortessa; BD Biosciences) with FACSDiva software (BD Biosciences). Data were analysed with FlowJo software version 10 (Tree Star, Inc., Ashland, OR, USA).

### Statistical analysis

Comparisons between unpaired nonparametric groups (Adult x Newborns) were performed with the Mann–Whitney test, and comparisons between paired nonparametric samples (ex: baseline and stimulated conditions) within the same group were performed with the Wilcoxon signed-rank test. The level of significance considered was p ≤ 0.05, using GraphPad Prism 7.

## Results

### High levels of CCL2 are induced by LPS in neonates

Although neonates exhibit an impaired IFN-γ response, they show hyperresponsiveness to cytokines induced by TLR activation^5^. Considering that bacteria can also secrete histamine and microbiota-derived histamine influences host immunological processes^8^, along with the broad modulatory effects exerted on immune responses by histamine^19^, we sought to evaluate the immunomodulatory function of histamine in the neonatal response to LPS. Chemokines are important mediators in immune cell recruitment and along with other factors lead orchestration of the inflammatory response. Accordingly, we first assessed chemokine secretion by MNCs from NBs (cord blood) induced by the TLR4 agonist LPS compared with their adult counterparts.

**Figure 1:**
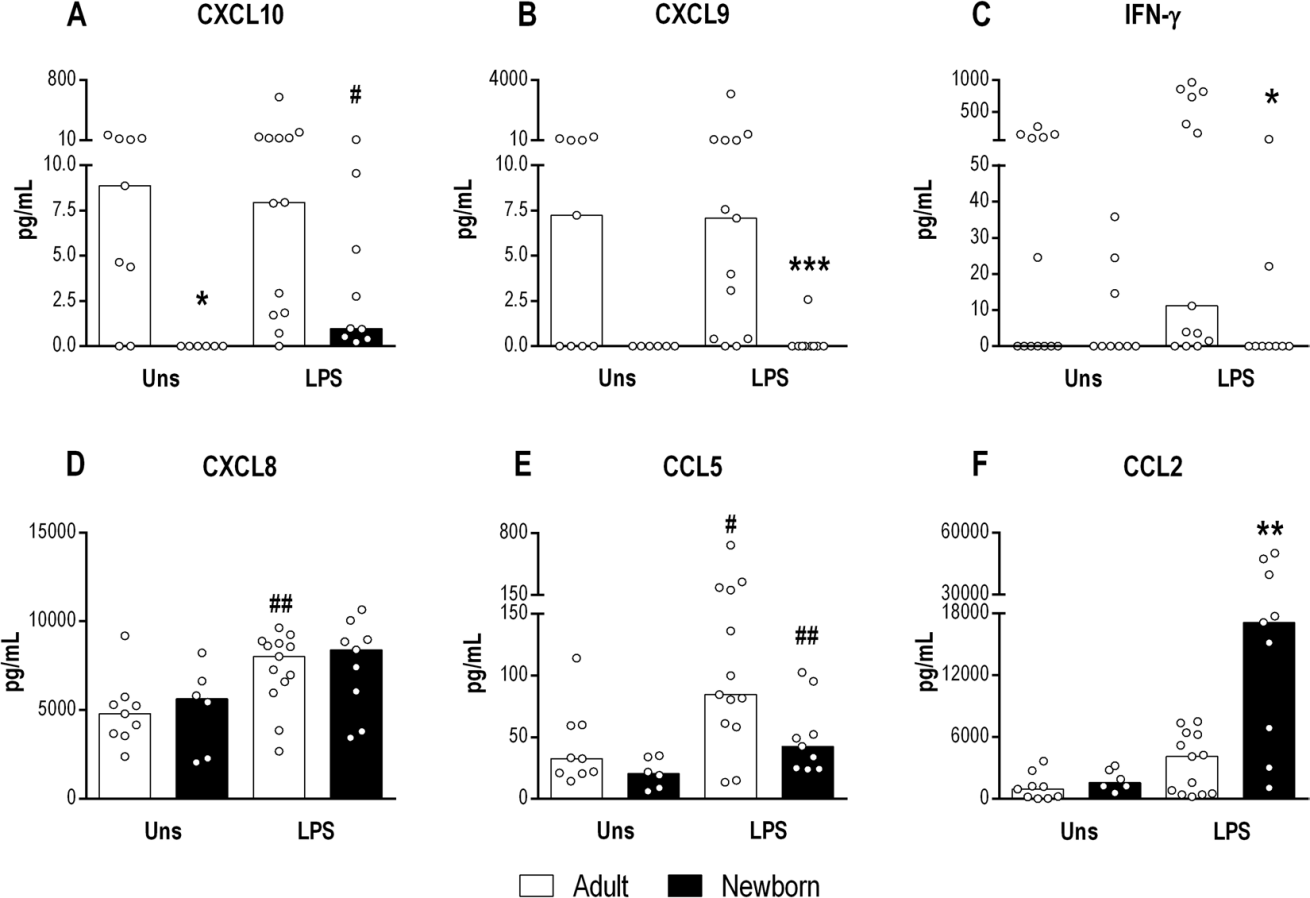
Impaired production of IFN-γ-inducible chemokines by MNCs of NBs in response to LPS. The presence of CXCL10 (**A**), CXCL9 (**B**), IFN-γ (**C**) CXCL8 (**D**), CCL5 (**E**) and CCL2 (**F**) was assessed in the supernatants of MNC cultures from healthy adult subjects (n = 13) and newborns (NBs; n = 9) stimulated with a TLR4 agonist (LPS - 1 µg/mL) or non-stimulated (Uns) for 24 h. Chemokines and cytokines were measured using a cytometric bead array and flow cytometry. Data are shown as the median. *p ≤ 0.05, **p ≤ 0.01,***p ≤ 0.001 compared with adults; ^#^p ≤ 0.05 and ^##^p ≤ 0.01 compared with baseline.

The levels of IFN-γ-dependent chemokines, such as CXCL10 and CXCL9, secreted by NB cells were considerably reduced compared with those secreted by adult cells (Fig. 1A,B). As these chemokines are induced by IFN-γ, we verified impairment of IFN-γ secretion caused by TLR4 activation in MNCs from NBs (Fig. 1C). Equivalent levels of other chemokines, such as CXCL8 and CCL5, after LPS stimulation were found in NB and adult cells (Fig. 1D,E). Notably, a 5-fold increase in CCL2 secretion upon TLR4 stimulation was detected in NB cells (Fig. 1F).

As we verified reduced CXCL10 and CXCL9 secretion induced by LPS in NB cells, we analyzed the effects of LPS stimulation in the presence of rIFN-γ (Fig. S1). We observed that rIFN-γ is able to restore chemokine secretion in NB cells to a level similar to that of adult.

We previously demonstrated the potential of the dual agonist TLR7/8 (CL097) to activate the immune response^20,21^; therefore, we evaluated the effect of TLR7/8 activation on NB MNCs. Curiously, CL097 partially restored CXCL10 (Fig. S2A) and CXCL9 (Fig. S2B) secretion in NB cells. Similar to the results for TLR4 stimulation, stimulation with CL097 resulted in decreased IFN-γ levels (Fig. S2C) but high levels of CCL2 (Fig. S2F) in NB cells compared with those of adults. Other cytokines, such as IL-10 and IL-12p70, were also induced by CL097 in adults, though this was not observed in NB cells (Fig. S3).

Enhanced CCL2 secretion, regardless of TLR4 or TLR7/8 activation, appears to be a feature of NB cells upon TLR activation.

### Histamine induces inhibitory effects on LPS-induced CCL2 secretion

**Figure 2:**
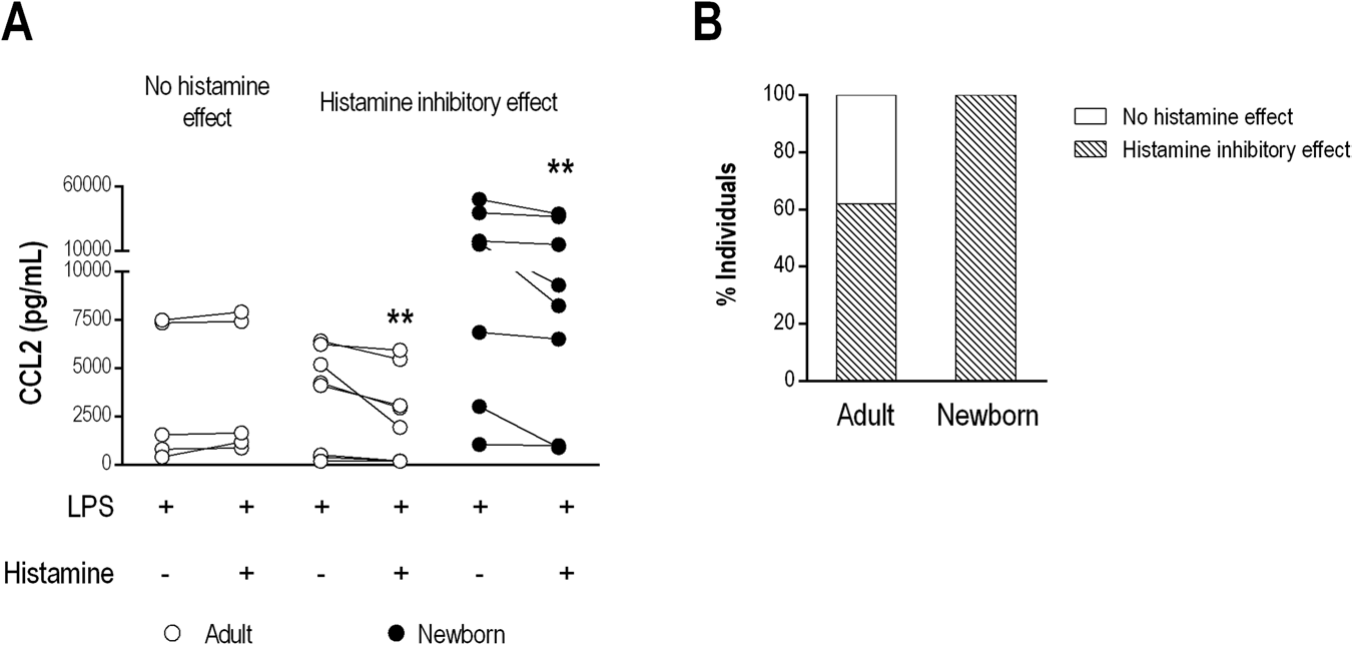
Histamine exerts an inhibitory effect on LPS-induced CCL2 production in NBs and adults. CCL2 production induced in MNCs from adults (n = 12) and NBs (n = 8) stimulated with LPS (1 µg/mL) in the presence of histamine (10 µM) for 24 h. CCL2 levels in the supernatants of the MNC cultures were measured using a cytometric bead array and flow cytometry. Individual data for histamine treatment of adult cells (open circle) showing no histamine effect (n = 5) or adult (n = 7) or NB (closed circle, n = 8) cells showing an inhibitory effect (**A**) and the frequency of the histamine inhibitory effect on CCL2 or no effect in newborns and adult cells (**B**). Data are shown as bars or before-after. **p ≤ 0.01 compared with LPS stimulation without histamine.

In addition to modulating macrophage polarization, CCL2 mediates monocyte egress from bone marrow and recruitment into inflamed tissues^22^. Accordingly, we next evaluated the effect of histamine on CCL2 secretion induced by a TLR4 agonist in MNCs from both NBs and adults. Interestingly, an inhibitory effect on LPS-induced CCL2 secretion mediated by histamine was observed in 100% of NBs and 65% of adults, with the remaining 35% of adult cells being refractory to the effects of histamine (Fig. 2A,B).

In adults, the absence of a histamine effect on CCL2 response modulation was not associated with symptomatic allergy; indeed, no rhinitis or asthma was present in these individuals. Therefore, to evaluate the effect of histamine on the LPS response, we further examined the adult cells showing an inhibitory effect in our subsequent analysis.

**Figure 3:**
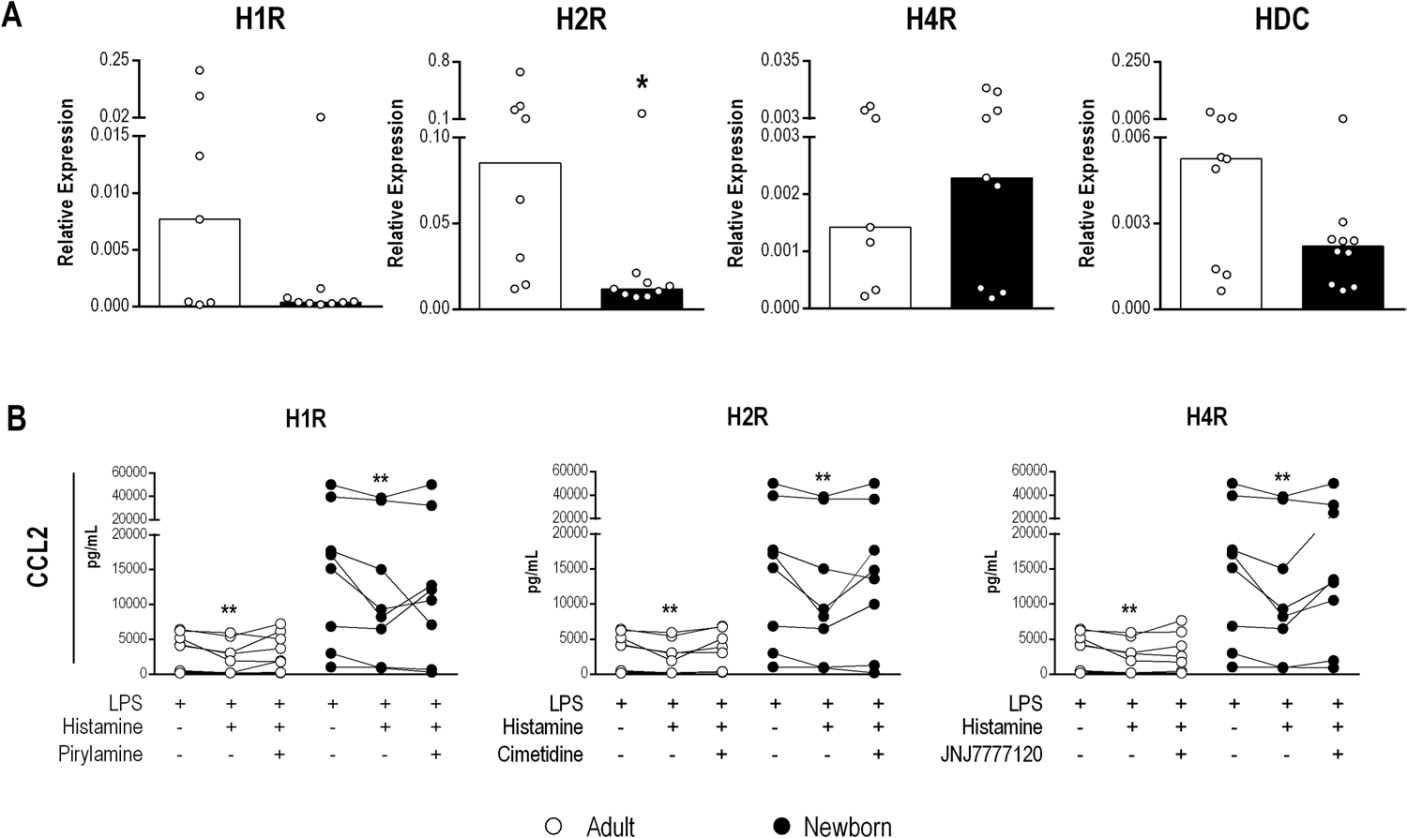
Participation of histamine receptors in the inhibitory effect on CCL2 production by LPS-stimulated MNCs from adults and newborns. Healthy adult (n = 7) and NB (n = 9) MNCs were analyzed for mRNA expression of HRs (H1R, H2R and H4R) and histidine decarboxylase (HDC) via qPCR normalized with GAPDH (**A**). Cultures of MNCs from healthy adults (n = 7) and NBs (n = 9) were incubated with histamine receptor antagonists of H1R (Pyrilamine −100 µM), H2R (Cimetidine - 1000 µM) and H4R (JNJ7777120 - 1 µM) for 1 h at 37 °C and thereafter with histamine (10 µM) and a TLR4 agonist (LPS - 1 µg/mL) for 24 h. The presence of CCL2 in the supernatant was measured using a cytometric bead array and flow cytometry (**B**). Data are shown as bars and before-after. *p ≤ 0.05 compared with adults; **p ≤ 0.01 compared with LPS stimulation.

The pleiotropic effects of histamine may be explained in part by the existence of four different receptors through which a mediator can signal. Analysis of the constitutive expression of HRs and histidine decarboxylase (HDC), the enzyme responsible for intracellular synthesis of histamine, revealed that H2R mRNA levels were lower in MNCs from NBs than in MNCs from adults (Fig. 3A). H3R expression was undetectable in MNCs from both adults and NBs. Next, to determine the influence of HRs involved in inhibition of CCL2, we treated MNCs with HR antagonists (pyrilamine, cimetidine and JNJ7777120 for blockade of H1R, H2R and H4R, respectively prior to LPS stimulation to assess CCL2 secretion in the presence of histamine (Fig. 3B). The effects of histamine on LPS stimulation were partially reversed by inhibition of H1R, H2R and H4R, demonstrating that the three HRs are required for histamine modulation in both NBs and adults.

### Histamine modulates the pro-inflammatory status of monocytes from neonates

**Figure 4:**
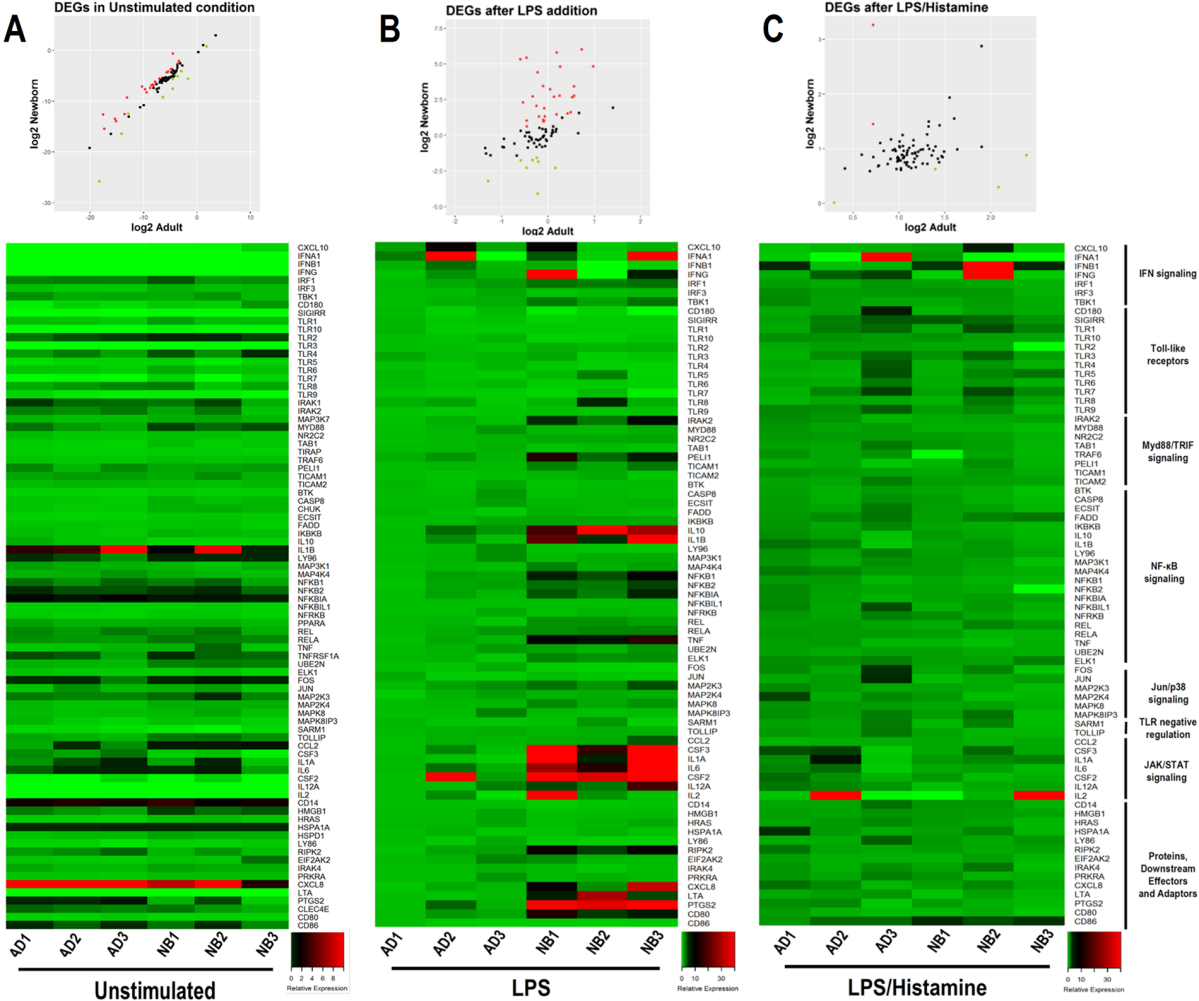
Histamine promotes down-regulation of overexpressed DEGs related to the TLR signaling pathway in NBs monocytes stimulated by LPS. Purified monocytes from healthy adults (ADs, n = 3) and NBs (n = 3) in an unstimulated condition (**A**), stimulated with LPS (1 µg/mL) (**B**) or LPS and histamine (10 µM) (**C**) for 4 h. A total of 84 genes related to TLR signaling were assessed by qPCR. DEGs of NBs relative to adults and a heat map of gene expression under unstimulated conditions normalized against 4 different internal genes (**A**) after LPS stimulation normalized against unstimulated (**B**) and after LPS/histamine stimulation normalized against LPS stimulation (**C**).

The reduced CCL2 response to TLR activation promoted by histamine treatment suggests that histamine can modulate factors involved in TLR signaling. To test this hypothesis, we applied a PCR array of 84 genes related to TLR pathways to purified monocytes. Monocytes were selected because they are the main mononuclear cell type that produces CCL2^23^. Unstimulated and LPS-stimulated monocytes from 3 adults and 3 NBs were assessed. Importantly, under unstimulated conditions, similar gene expression profiles were observed for adult and NB monocytes (Fig. 4A). These data are also illustrated in Volcano plot and Denn diagram (Fig. S4).

Upon LPS stimulation, NB monocytes showed up-regulation of genes IL-1A, IL-1B, IL-10, TNF, IL-6, and IL-2, which are related to the JAK/STAT/NF-kB and IFN pathways (Fig. 4B). Overexpression of TLR signaling genes in NBs is a physiological response during pregnancy, which suggests a mechanism for promoting protection against bacterial infections. Moreover, up-regulation of proinflammatory cytokine genes, such as CCL2, IL-1β and IL-6, was associated with high protein levels in NB serum compared with adult levels (Fig. S5).

**Figure 5:**
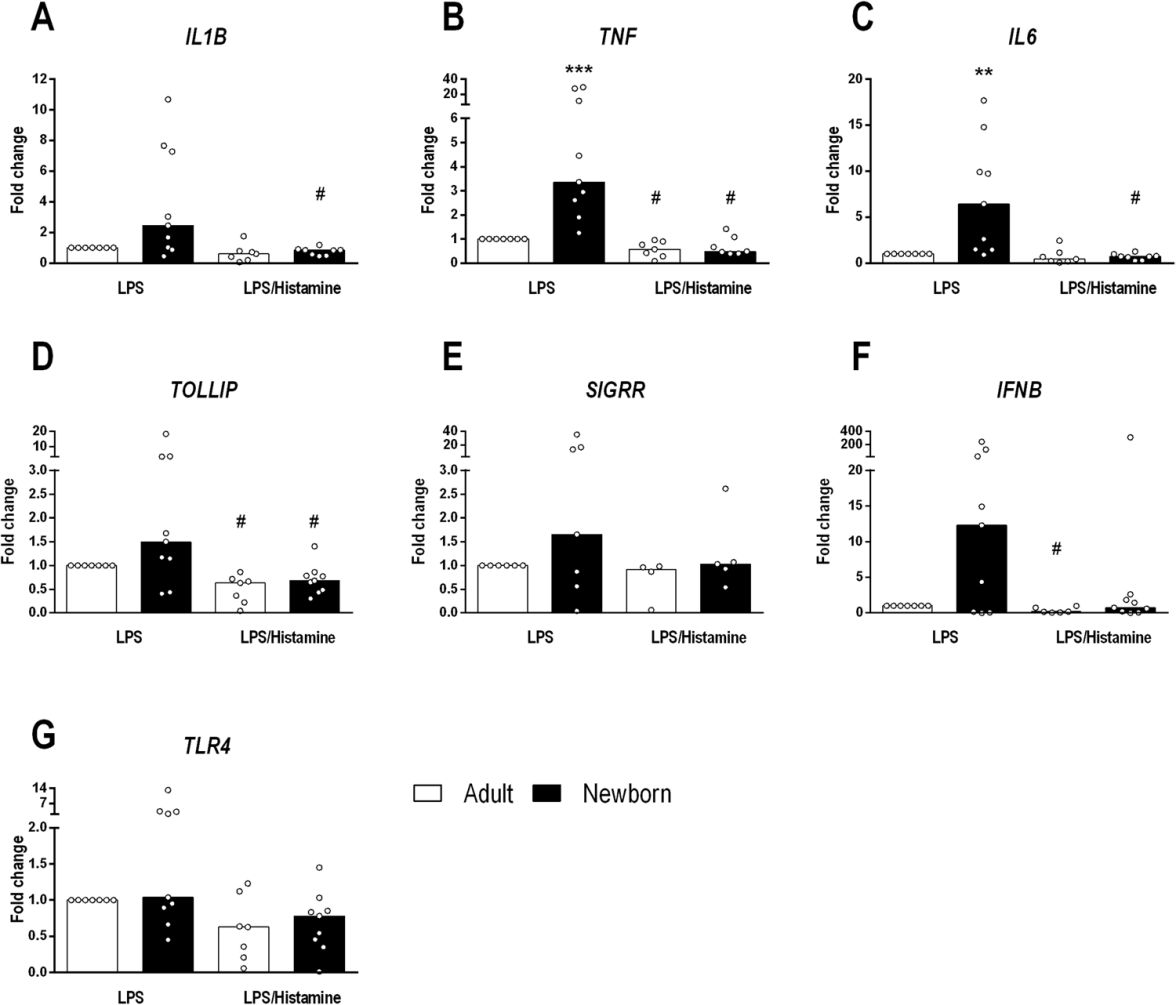
Up-regulation of transcripts of cytokines and regulatory factors in NBs is down-modulated by histamine. Purified monocytes from healthy adults (n = 7) and NBs (n = 9) were stimulated with LPS (1 µg/mL) or LPS plus histamine (10 µM) for 4 h. mRNA expression of IL1B (**A**), TNF (**B**), IL6 (**C**), TOLLIP (**D**), SIGRR (**E**), IFNB (**F**) and TLR4 (**G**) after LPS stimulation or LPS plus histamine stimulation was assessed by qPCR and normalized against LPS stimulation of adult cells. Data are shown as the median, **p ≤ 0.01 and ***p ≤ 0.001 compared with adults.; ^#^p ≤ 0.05 and ^##^p ≤ 0.01 compared with LPS stimulation.

**Figure 6:**
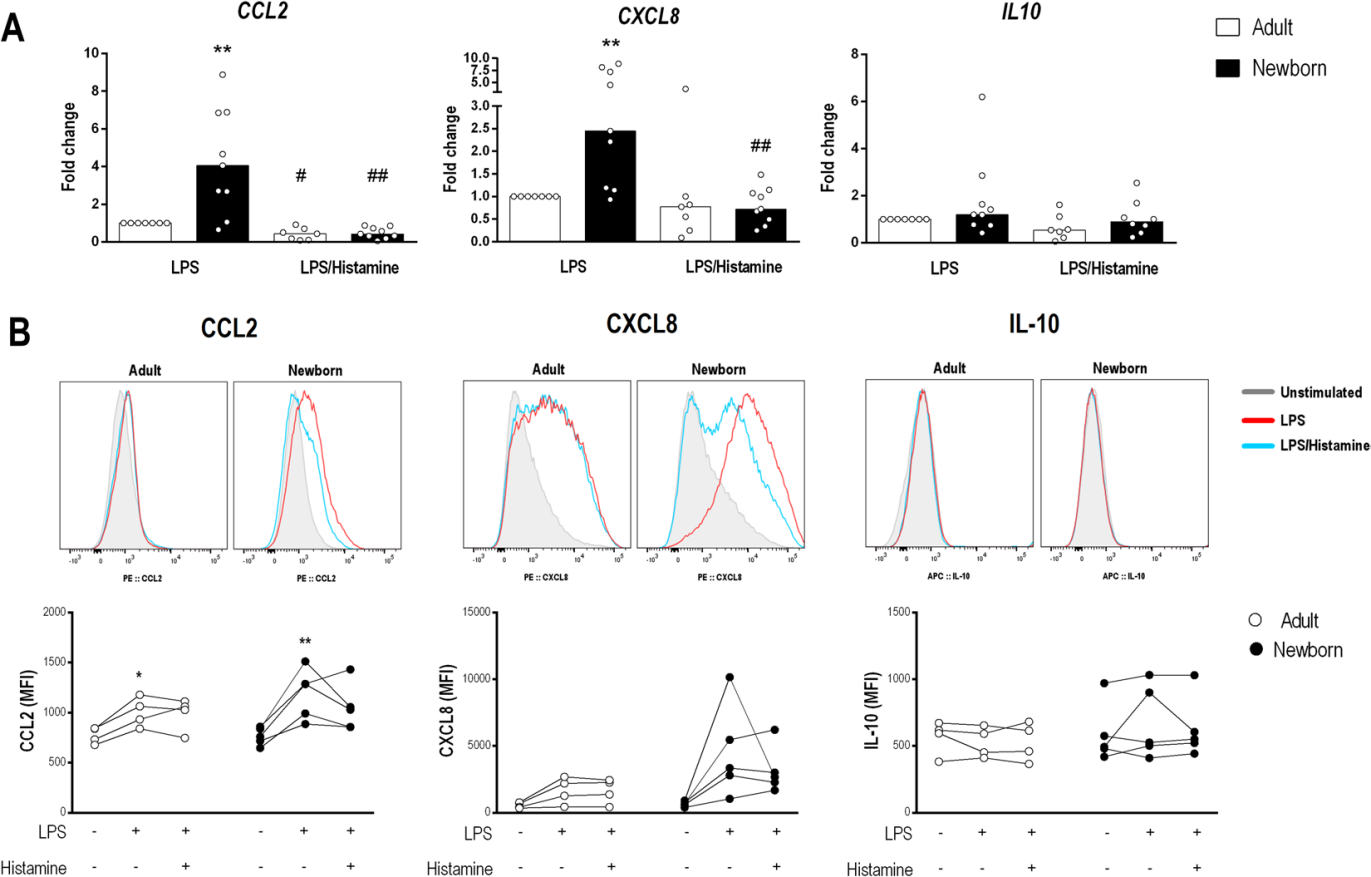
Histamine down-regulates CCL2 and CXCL8 genes and protein levels in NB monocytes stimulated by LPS. Purified monocytes from healthy adults (n = 7) and NBs (n = 9) were stimulated with LPS (1 µg/mL – red line) or LPS plus histamine (10 µM – blue line) or non-stimulated (Uns – gray line) for 4 h. mRNA expression of CCL2, CXCL8 and IL10 (**A**) was assessed by qPCR and normalized against LPS stimulation of adult cells. Data are shown as the median ** p ≤ 0.01 compared with adults; ^#^p ≤ 0.05 and ^##^p ≤ 0.01 compared with LPS stimulation. (**B**) Purified monocytes from healthy adults (n = 4) and NBs (n = 5) were stimulated with LPS or LPS plus histamine for 18 h, and intracellular staining for CCL2, CXCL8 and IL-10 was assessed by flow cytometry. Data show the median fluorescence intensity (MFI). *p ≤ 0.05, **p ≤ 0.01 compared with baseline.

Based on the PCR array data, we performed quantitative PCR (q-PCR) on some target genes to verify the histamine modulatory effect (Fig. 5). Strikingly, most of the overexpressed genes induced by LPS in NBs were down-regulated by histamine, resulting in a similar profile in adults and NBs (Fig. 5). Next, we assessed monocyte intracellular chemokine/cytokine expression by flow cytometry; the gating strategy is shown in Fig. S6. According to the transcript levels of CCL2 and CXCL8 in monocytes (Fig. 6A), histamine exerted a modulatory effect of a decreasing trend, which was also detected at the protein level, as verified by the mean fluorescence intensity of intracellular CCL2 and CXCL8 levels (Fig. 6B). Levels of other cytokines, such as IL-10, were unchanged by histamine. The down-modulatory effect of histamine on differentially expressed genes (DEGs) was more evident in neonates than in adults, as verified by the 100% inhibitory function exerted by histamine on CCL2 secretion (Fig. 2).

The findings of the present study demonstrate the immunomodulatory effect of histamine on TLR signaling genes in LPS-activated monocytes from NBs.

## Discussion

The balance between inflammatory and regulatory factors induced by TLR stimulation, including establishment of the neonatal microbiota and bacterial infections, is crucial for controlling inflammation during the early stages of life. The regulatory effects of histamine are the result of a complex network comprising the types of cells involved, cross-talk among different HRs, and the distinct intracellular signals generated^24^. Our work identifies histamine as a player in immune homeostasis during the neonatal period that can counterbalance the hyperresponsiveness of NBs to TLR stimulation.

Neonatal innate immune reactivity has been shown to occur in response to TLR activation, triggering the production of high levels of proinflammatory cytokines, despite impairment of some others such as IFN-γ^5,25^. Compared with adult MNCs, IFN-γ-inducible chemokines, such as CXCL10 and CXCL9, were barely detectable in MNCs from NBs activated by LPS. The main cellular source of these chemokines in MNCs from NBs is monocytes, and TLR4 expression in NB monocytes was similar to that in adult monocytes. This finding suggests that the changes in IFN-induced chemokines observed in MNCs were due to impaired IFN-γ and IFN type I secretion, which may in turn influence these IFN-γ-inducible chemokines. In fact, low IFN-γ levels were detected following both TLR4 and TLR7/8 agonist activation in NB cells, indicating involvement of the TLR pathway. CXCL10 secretion has been reported to be impaired in neonatal blood cells upon LPS challenge in association with impaired expression of IFNβ and IFN-inducible genes^26^. Moreover, phosphorylated IRF3 may translocate to the nucleus, inducing expression of the *IFNB* gene as well as other genes, such as CXCL10^27^. TRIF-dependent activation of IRF3 downstream of TLR3 or TLR4 is compromised in neonatal DCs.

Imiquimod (TLR7 agonist) induces Th1-type responses by neonatal APCs exceeding those induced by other TLR agonists via TLR8- and caspase-1-mediated pathways that are refractory to the inhibitory effects of adenosine/cAMP^28^. In our study system, the dual TLR7/TLR8 agonist did not induce IFN-γ but partially restored IFN-γ-inducible chemokines in NB cells. TLR4 activates both MyD88-dependent and TRIF-dependent pathways, and CXCL10 is TRIF/TRAM dependent, which suggests that the MyD88-independent TLR4 pathway is affected in NB cells.

In contrast to IFN-γ-inducible chemokines, CCL2 was detected at higher levels in MNCs and serum from NBs than the levels verified in adults. These CCL2 levels are characteristic of neonates and not due to the parturition process, as we previously found that full-term healthy mothers exhibit lower serum CCL2 levels than do neonates^29^. Decreased expression of proinflammatory factors, including CCL2, has been reported on healthy pregnancies^30^. The increased fetal levels of CCL2 may be influenced by the placental microenvironment. The CCL2/CCR2 axis may regulate the biological functions of endometrial stromal cells (ESCs) via NF-κB and ERK1/2 pathways^31^, and estrogen may stimulate thymic stromal lymphopoietin, which can induce CCL2 secretion by ESCs, both of which can drive the Th2 response. Importantly, in all the chemokines evaluated, it was seen a dispersion in the cytokine production, induced by TLR4 activation. It is possible that the maternal metabolic status as well as the vaccination history of the pregnant women influences the level of chemokines evaluated in the mononuclear cells of the newborns.

Taking into account the proinflammatory status of neonates, as based on CCL2 responsiveness following LPS activation, we evaluated whether histamine plays a modulatory role. Histamine was able to down-regulate CCL2 secretion by MNCs, an inhibitory effect that was more prominent in NB cells. The lower H2R expression found in NB MNCs may favor the inflammatory response because H2R positively interferes with the peripheral antigen tolerance induced by regulatory T cells and decreases CXCL10, IL-12 and TNF-α levels while increasing IL-10 secretion induced by LPS in monocyte-derived DCs from adult subjects^32^. Differential effects of histamine have been verified in other cell types, such as a down-regulatory effect exerted by H4R on CCL2 secretion in monocytes from adults^33^. We verified the partial involvement of H1R, H2R and H4R in mediating the inhibitory effect on CCL2 secretion in both neonates and adults. Participation of the three HRs in the modulatory histamine effect may have occurred because we used total MNCs rather than a purified population. We were not able to detect H3R in MNCs in our q-PCR analyses, even though H3R expression in DCs has been reported^34^.

Exogenous histamine down-regulated CCL2, demonstrating its modulatory effect on TLR activation. In view of the modulatory role of histamine, we evaluated expression of genes related to NF-κB, JAK/STAT, MyD88/TRIF and TLR signaling in monocytes. This profiling of genes involved in TLR signaling revealed an abundance of DEGs in LPS-activated monocytes from NBs. In association with the observed up-regulation of CCL2 and CXCL8 genes in LPS-activated monocytes induced by LPS in neonates, histamine was able to decrease the levels of these cytokines at both transcript and protein levels, suggesting that the histamine modulatory effect occurred at transcriptional level. Histamine suppression in DCs of histamine receptor 2 (H2R)-deficient mice upon LPS activation was due to H2R signaling through cAMP and an exchange protein directly activated by cAMP^32^. Regardless, further investigation is required to determine how histamine may interfere with chemokine secretion.

Moreover, the IL-1B, TNF and IL-6 genes, expression of which was pronounced in NB monocytes upon LPS stimulation, were down-regulated by histamine. These protein were founded at high levels in serum, and it remains to be further explored whether this increased serum level of IL-1β protein in NBs indicates inflammasome NLRP3 activation. The effects of adding exogenous histamine may suggest that *in vivo* histamine may exert a versatile dual function, sometimes inhibiting or stimulating the inflammatory response, such as in allergy.

Other mechanisms may contribute to the control of the inflammatory response, including epigenetics and post-transcriptional regulation. Differentially expressed microRNAs have been identified in cord blood-derived leukocytes under conditions of LPS-induced acute inflammation^35^. Understanding the negative regulation of these pathways is crucial for furthering our understanding of how to control inflammation in the neonatal period.

It was notable that most of the overexpressed and moderately expressed genes induced by LPS in NBs were down-regulated by histamine. Levels of proinflammatory molecules such as IL-6, TNF, IFNB and IL-1B were reduced by histamine in monocytes from NBs. These data reveal a differential pattern between NBs and adults, showing that NB cells are more susceptible to the negative regulatory effect of histamine, even expressing lower constitutive H2R levels than adult cells. It is possible that NB cells are more susceptible to the regulatory effects of histamine because they are prone to innate sensing of inflammatory genes under the effects of LPS signaling.

Altogether, the data presented herein demonstrate that histamine has a modulatory function in the LPS-induced inflammatory response in NB monocytes. This function may avoid excessive inflammation in early life, even in microbiota colonization or bacterial infection. Together with other regulatory factors or cells mediating regulatory functions, histamine can play an immunoregulatory role as well as a role in immune homeostasis at the maternal-fetal interface, including during the neonatal period.

## Supplementary information

Supplementary Information
