# Supplementary material for: Proinflammatory profile of neonatal monocytes induced by microbial ligands is downmodulated by histamine
Source: Sci Rep. 2019 Sep 23;9:13721. doi: 10.1038/s41598-019-50227-8 (PMC6757139; doi:10.1038/s41598-019-50227-8)
Supplement: Supplementary file 1 — Supplementary Information [file 41598_2019_50227_MOESM1_ESM.pdf]

Supplementary Information

Proinflammatory profile of neonatal monocytes induced by microbial ligands is downmodulated by histamine

Anna Cláudia Calvielli Castelo Branco[1]; Nátalli Zanete Pereira[1]; Fábio Seiti Yamada Yoshikawa[1]; Luanda Mara da Silva Oliveira[1]; Franciane Mouradian Emidio Teixeira[1]; Luana de Mendonça Oliveira[1]; Anna Julia Pietrobon[1]; Marina Passos Torrealba[1]; Josenilson Feitosa de Lima[1]; Alberto José da Silva Duarte[1]; Maria Notomi Sato[1].

[1]Laboratory of Medical Investigation, LIM-56, Department of Dermatology, Tropical Medicine Institute of São Paulo, Medical School , University of São Paulo, Brazil.

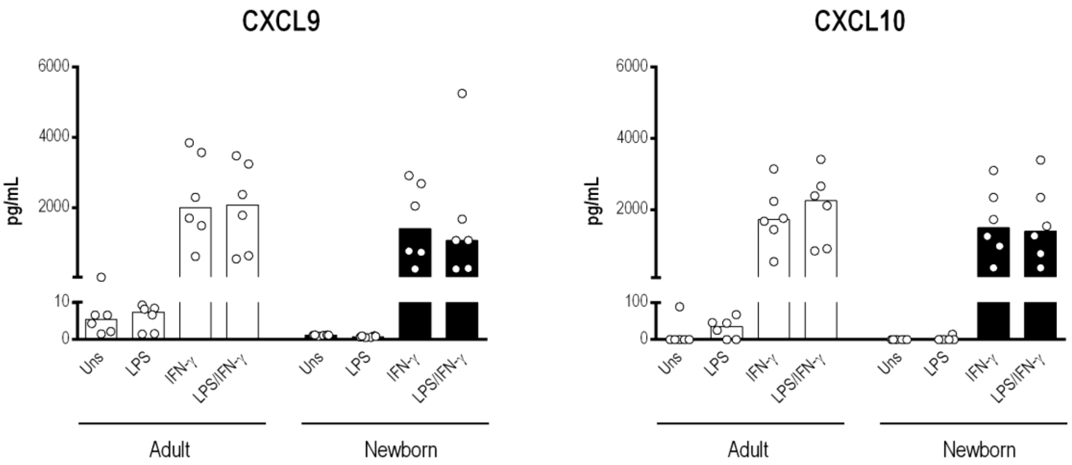

Figure S1. IFN-γ restores the production of CXCL9 and CXCL10 induced by LPS in adults and NBs. The presence of CXCL9 and CXCL10 was assessed in the culture supernatants of MNCs from healthy adult subjects (n=6) and newborns (NBs; n=6) stimulated with a TLR4 agonist (LPS - 1µg/mL) and IFN-γ (25ng/mL) for 24 h. Chemokines were measured using a cytometric bead array and flow cytometry. Data are shown as the median.

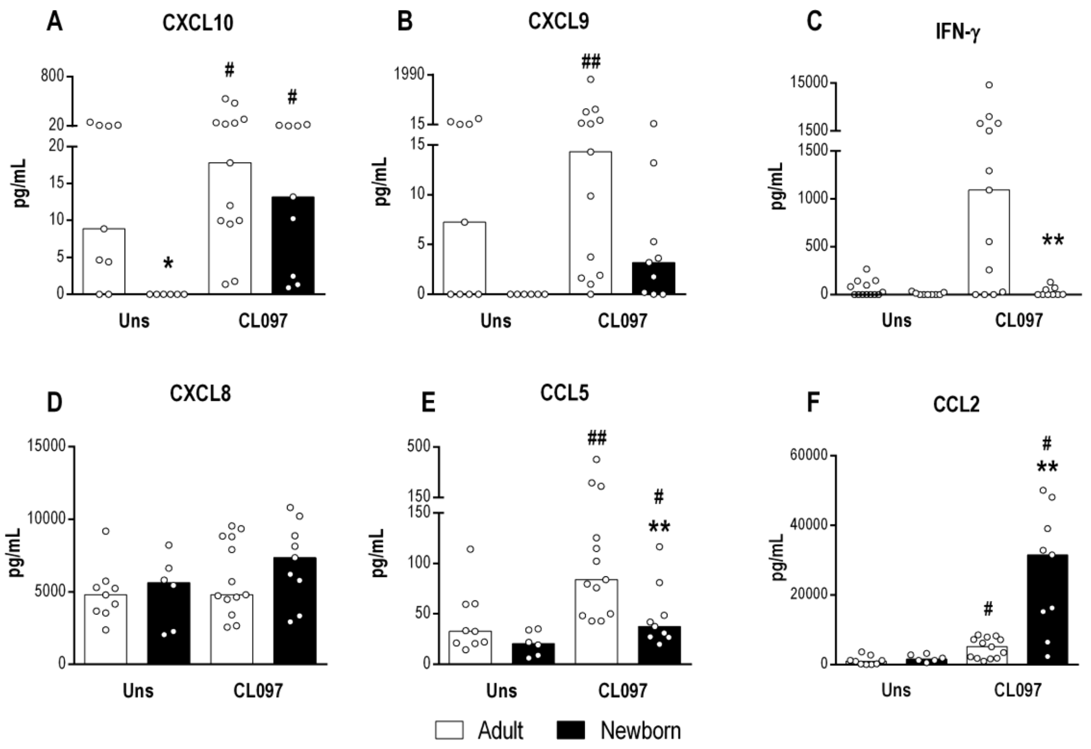

Figure S2. Up-regulation of CCL2 secretion upon CL097 stimulation in MNCs from NBs. MNCs from healthy adult subjects (n=13) and NBs (NBs; n=9) were incubated with a TLR7/8 agonist (CL097 – 2.5µg/mL) for 24 h. The presence of CXCL10 (A), CXCL9 (B), IFN-γ (C), CXCL8 (D), CCL5 (E) and CCL2 (F) in the cell supernatant was assessed using a cytometric bead array and flow cytometry. Data are shown as the median. *p≤0.05 and **p≤0.01 compared with adults; #p≤0.05 and ##p≤0.01 compared with baseline.

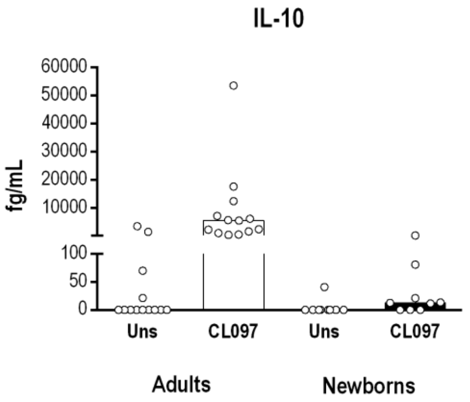

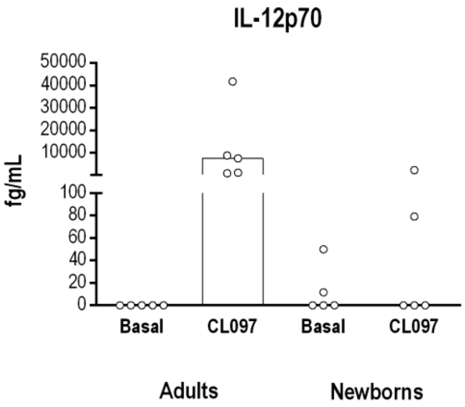

Figure S3. Impaired production of IL-10 and IL-12p70 by NBs after TLR7/8 stimulation. The presence of IL-10 and IL-12p70 was assessed in the culture supernatants of MNCs from healthy adult subjects (n=5-13) and newborns (NBs; n=5-9) stimulated with dual TLR7/8 agonist (CL097 – 2.5µg/mL) for 24 h. Cytokines were measured using a cytometric bead array and flow cytometry. Data are shown as the median.

A)

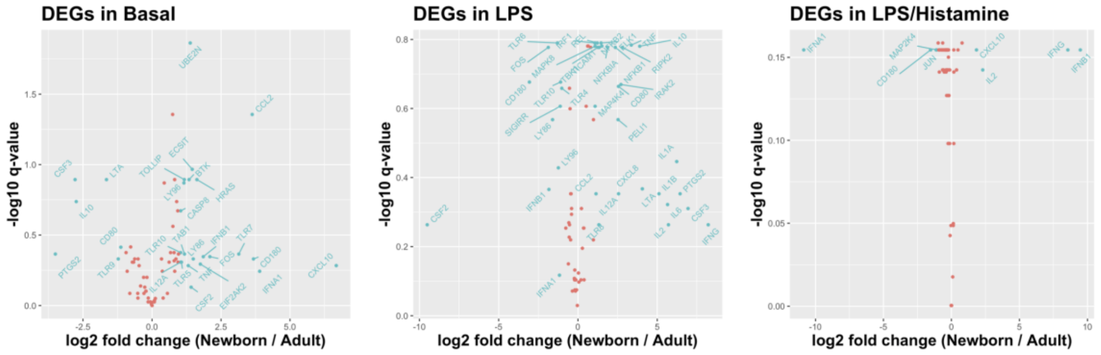

B)

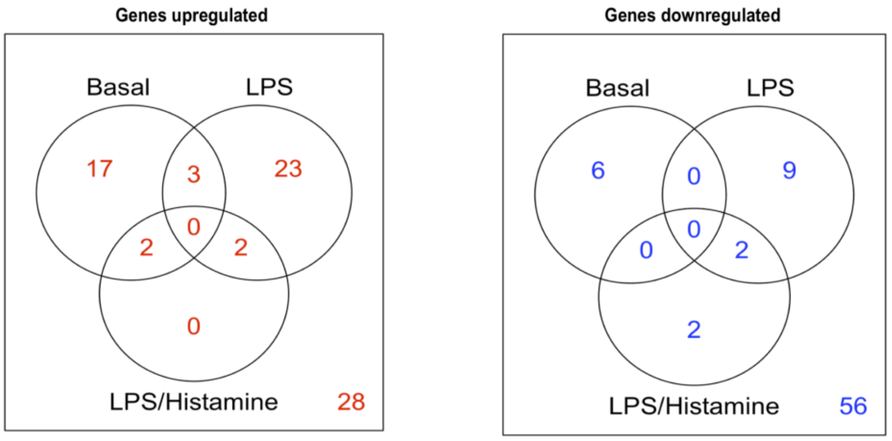

Figure S4. Histamine promotes down-regulation of overexpressed DEGs related to the TLR signaling pathway in NBs monocytes stimulated by LPS. Purified monocytes from healthy adults (ADs, n=3) and NBs (n=3) in an unstimulated condition , stimulated with LPS (1µg/mL) or LPS and histamine (10µM) for 4 h. A total of 84 genes related to TLR signaling were assessed by qPCR. DEGs of NBs relative to adults are illustrated in Volcano plot (A) and Denn diagram (B).

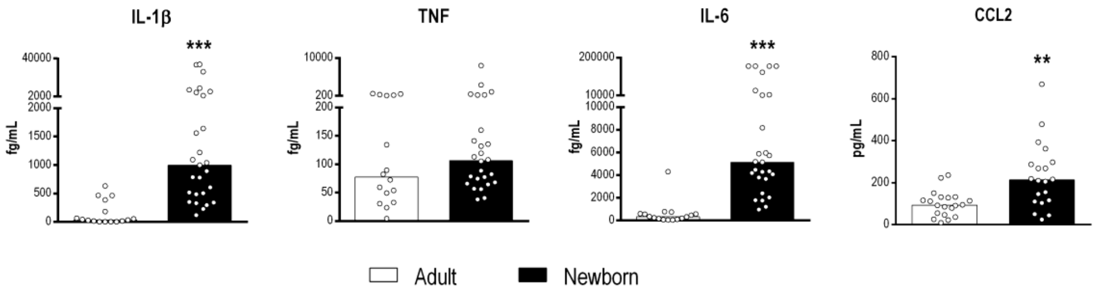

Figure S5. High levels of proinflammatory cytokines in NB serum. The presence of IL-1β, TNF, IL-6 and CCL2 was assessed in the serum of healthy adults (n=16-20) and NBs (n=20-27) using a cytometric bead array and flow cytometry. Data are shown as the median. **p≤0.01 and ***p≤0.001 compared with adults.

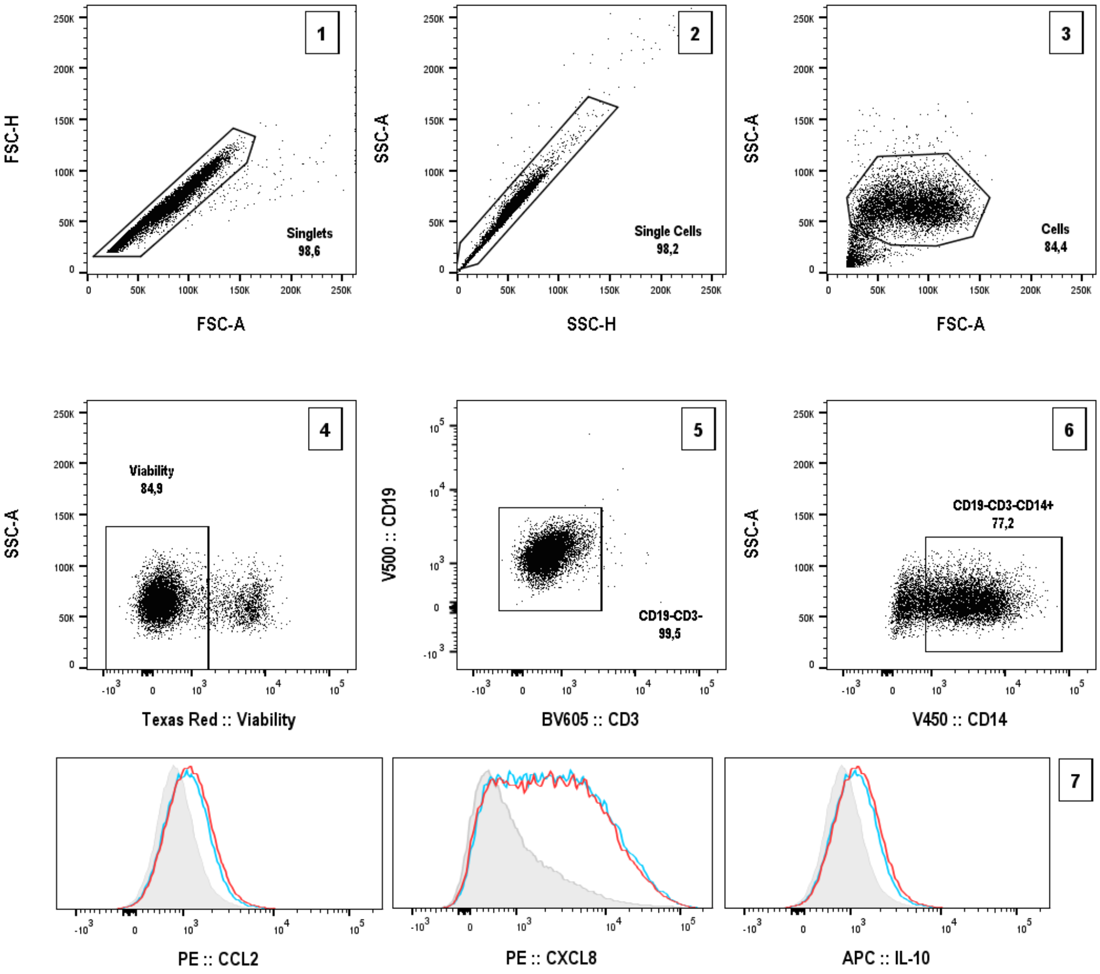

Figure S6. Gating strategy for analysis of CCL2, CXCL8 and IL-10 in monocytes. Primary monocytes were stimulated with LPS (1µg/mL) and/or histamine (10µM) in the presence of Brefeldin A (10µg/mL) for 18 h, and chemokines/cytokines were labeled by intracellular staining. Singlets (forward scatter (FSC) and side scatter (SSC), gates 1 and 2, respectively), followed by total (gate 3) and live (gate 4) cells, were selected. Within this population, CD3-/CD19-/CD14+ cells (gate 5 and 6) were examined for expression (MFI) of CCL2 or CXCL8 (PE) and IL-10 (APC) (gate 7).

Table S1. Oligonucleotides used for qPCR amplification

Primer	Sequence
H1R	5’- TCTCGAACGGACTCAGATACCA- 3’(forward)<br>5’- CCTGTGTTAGACCCACTCCTCAA- 3’(reverse)
H2R	5’- TACCAGCTGTCCTGCAAGTG- 3’ (forward)<br>5’- CCCCAGGTGGATAGA CAG AA- 3’ (reverse)
H3R	5'- TCGTGCTCATCAGCTACGAC- 3' (forward)<br>5'- AAGCCGTGATGAGGAAGTAC- 3' (reverse)
H4R	5'- GGCTCACTACTGACTATCTG- 3' (forward)<br>5'- CCTTCATCCTTCCAAGACTC- 3' (reverse)
HDC	5’- TTGATTGCCCTGCTGGCAGC- 3’ (forward)<br>5’- TGCACAGACAAAGACGGGCACC- 3’ (reverse)
IL1B	5’- TCCCCAGCCCTTTTGTTGA -3’ (forward)<br>5’- TTAGAACCAAATGTGGCCGTG -3’ (reverse)
TNF	5´-CCCAGGCAGTCAGATCATCTTC-3´ (forward)<br>5´-GCTTGAGGGTTTGCTACAACAT-3´ (reverse)
IL6	5’- CCTGAGAAAGGAGACATGTAA -3’ (forward)<br>5’- GGCAAGTCTCCTCATTGAATCC -3’ (reverse)
TOLLIP	5´-CTCTCCTTCTCATGCCGTTC-3´ (forward)<br>5´-GTGTGAGGGATTGTGTGTGC-3´ (reverse)
SIGRR	5´-CCATGCCAGGTGTCTGTGAT-3´ (forward)<br>5´-CGTCTTTCAGCCACTGGACT-3´ (reverse)
IFNB	5’- CATTACCTGAAGGCCAAGGA-3’ (forward)<br>5’-CCATTGTCCAGTCCCAGAGG -3’ (reverse)
TLR4	5′- CAGAGTTTCCTGCAATGGATCA- 3′ (forward)<br>5′- GCTTATCTGAAGGTGTTGCACA- 3′ (reverse)
GAPDH	5′- GAA GGT GAA GGT CGG AGT- 3′ (forward)<br>5′- GAA GAT GGT GAT GGG ATT TC- 3′ (reverse)

Table S2. TLR signaling pathway genes analyzed by PCR array

Symbol	Description	Symbol	Description
BTK	Bruton agammaglobulinemia tyrosine kinase	MAP2K4	Mitogen-activated protein kinase 4
CASP8	Caspase 8, apoptosis-related cysteine peptidase	MAP3K1	Mitogen-activated protein kinase 1
CCL2	Chemokine (C-C motif) ligand 2	MAP3K7	Mitogen-activated protein kinase 7
CD14	CD14 molecule	MAP4K4	Mitogen-activated protein kinase 4
CD180	CD180 molecule	MAPK8	Mitogen-activated protein kinase 8
CD80	CD80 molecule	MAPK8IP3	Mitogen-activated protein kinase 8 interacting protein 3
CD86	CD86 molecule	MYD88	Myeloid differentiation primary response gene (88)
CHUK	Conserved helix-loop-helix ubiquitous kinase	NFKB1	Nuclear factor of kappa light polypeptide gene enhancer in B-cells 1
CLEC4E	C-type lectin domain family 4, member E	NFKB2	Nuclear factor of kappa light polypeptide gene enhancer in B-cells 2 (p49/p100)
CSF2	Colony stimulating factor 2 (granulocyte-macrophage)	NFKBIA	Nuclear factor of kappa light polypeptide gene enhancer in B-cells inhibitor, alpha
CSF3	Colony stimulating factor 3 (granulocyte)	NFKBIL1	Nuclear factor of kappa light polypeptide gene enhancer in B-cells inhibitor-like 1
CXCL10	Chemokine (C-X-C motif) ligand 10	NFRKB	Nuclear factor related to kappaB binding protein
ECSIT	ECSIT homolog (Drosophila)	NR2C2	Nuclear receptor subfamily 2, group C, member 2
EIF2AK2	Eukaryotic translation initiation factor 2-alpha kinase 2	PELI1	Pellino homolog 1 (Drosophila)
ELK1	ELK1, member of ETS oncogene family	PPARA	Peroxisome proliferator-activated receptor alpha
FADD	Fas (TNFRSF6)-associated via death domain	PRKRA	Protein kinase, interferon-inducible double stranded RNA dependent activator
FOS	FBJ murine osteosarcoma viral oncogene homolog	PTGS2	Prostaglandin-endoperoxide synthase 2 (prostaglandin G/H synthase and cyclooxygenase)
HMGB1	High mobility group box 1	REL	V-rel reticuloendotheliosis viral oncogene homolog (avian)
HRAS	V-Ha-ras Harvey rat sarcoma viral oncogene homolog	RELA	V-rel reticuloendotheliosis viral oncogene homolog A (avian)
HSPA1A	Heat shock 70kDa protein 1A	RIPK2	Receptor-interacting serine-threonine kinase 2
HSPD1	Heat shock 60kDa protein 1 (chaperonin)	SARM1	Sterile alpha and TIR motif containing 1
IFNA1	Interferon, alpha 1	SIGIRR	Single immunoglobulin and toll-interleukin 1 receptor (TIR) domain
IFNB1	Interferon, beta 1, fibroblast	TAB1	TGF-beta activated kinase 1/MAP3K7 binding protein 1
IFNG	Interferon, gamma	TBK1	TANK-binding kinase 1
IKBKB	Inhibitor of kappa light polypeptide gene enhancer in B-cells, kinase beta	TICAM1	Toll-like receptor adaptor molecule 1
IL10	Interleukin 10	TICAM2	Toll-like receptor adaptor molecule 2
IL12A	Interleukin 12A (natural killer cell stimulatory factor 1, cytotoxic lymphocyte maturation factor 1, p35)	TIRAP	Toll-interleukin 1 receptor (TIR) domain containing adaptor protein
IL1A	Interleukin 1, alpha	TLR1	Toll-like receptor 1
IL1B	Interleukin 1, beta	TLR10	Toll-like receptor 10
IL2	Interleukin 2	TLR2	Toll-like receptor 2
IL6	Interleukin 6 (interferon, beta 2)	TLR3	Toll-like receptor 3
CXCL8	Interleukin 8	TLR4	Toll-like receptor 4
IRAK1	Interleukin-1 receptor-associated kinase 1	TLR5	Toll-like receptor 5
IRAK2	Interleukin-1 receptor-associated kinase 2	TLR6	Toll-like receptor 6
IRAK4	Interleukin-1 receptor-associated kinase 4	TLR7	Toll-like receptor 7
IRF1	Interferon regulatory factor 1	TLR8	Toll-like receptor 8
IRF3	Interferon regulatory factor 3	TLR9	Toll-like receptor 9
JUN	Jun proto-oncogene	TNF	Tumor necrosis factor
LTA	Lymphotoxin alpha (TNF superfamily, member 1)	TNFRSF1A	Tumor necrosis factor receptor superfamily, member 1A
LY86	Lymphocyte antigen 86	TOLLIP	Toll interacting protein
LY96	Lymphocyte antigen 96	TRAF6	TNF receptor-associated factor 6
MAP2K3	Mitogen-activated protein kinase 3	UBE2N	Ubiquitin-conjugating enzyme E2N
